# Phagophores originate from endoplasmic reticulum membranes in vasopressin neurons in a mouse model of familial neurohypophysial diabetes insipidus

**DOI:** 10.1007/s00441-025-04013-w

**Published:** 2025-10-04

**Authors:** Takashi Miyata, Daisuke Hagiwara, Ryosei Ashida, Satoshi Naito, Yohei Kawaguchi, Tomoko Handa, Tomoko Kobayashi, Mariko Sugiyama, Takeshi Onoue, Shintaro Iwama, Hidetaka Suga, Ryoichi Banno, Mami Matsumoto, Hidetoshi Urakubo, Nobuhiko Ohno, Hiroshi Arima

**Affiliations:** 1 Department of Endocrinology and Diabetes, Nagoya University Graduate School of Medicine, 65 Tsurumai-Cho, Showa-Ku, Nagoya, 466-8550 Japan; 2 Department of Clinical Research Education, Nagoya University Graduate School of Medicine, Nagoya, 466-8550 Japan; 3 Physical Fitness and Sports, Research Center of Health, Nagoya University, Nagoya, 464-8601 Japan; 4 Section of Electron Microscopy, Supportive Center for Brain Research, National Institute for Physiological Sciences, Okazaki, 444-8787 Japan; 5 Department of Developmental and Regenerative Neurobiology Institute of Brain Science, Nagoya City University Graduate School of Medical Sciences, Nagoya, 467-8601 Japan; 6 Department of Biomedical Data Science, School of Medicine, Fujita Health University, Toyoake, 470-1192 Japan; 7 Division of Ultrastructural Research, National Institute for Physiological Sciences, Okazaki, 444-8787 Japan; 8 Department of Anatomy, Division of Histology and Cell Biology, School of Medicine, Jichi Medical University, Shimotsuke, 329-0498 Japan

**Keywords:** Arginine vasopressin (AVP), Endoplasmic reticulum (ER), ER-phagy, Familial neurohypophysial diabetes insipidus (FNDI), Phagophore, Serial block-face scanning electron microscopy (SBF-SEM)

## Abstract

Familial neurohypophysial diabetes insipidus (FNDI) is an autosomal dominant disorder caused by mutations in the arginine vasopressin (AVP) gene. In AVP neurons in a mouse model of FNDI, aggregates of mutant AVP precursors accumulate within a specific compartment of the endoplasmic reticulum (ER). However, as FNDI mice aged, or were exposed to repeated water deprivation, the ER lumen dilated and mutant aggregates dispersed throughout the ER. Meanwhile, autophagic isolation membranes, known as phagophores, emerged to envelop ER containing these aggregates, indicating induction of ER-phagy. Previous in vitro studies showed that phagophores originate from ER membranes, but the structural relationship between phagophores and the ER membrane in vivo remains unknown. In this study, we used serial block-face scanning electron microscopy to investigate the structural relationship between phagophores, ER membranes, and protein aggregates within dilated ER of AVP neurons from FNDI mice subjected to intermittent water deprivation for 4 weeks. Three-dimensional analysis revealed that phagophores enveloped aggregates located within the dilated ER. Serial imaging further demonstrated a physical connection between these phagophores and intact ER membranes. This study provides the first in vivo evidence of the structural continuity between phagophores and the ER membrane in AVP neurons in a mouse model of FNDI.

**Supplementary Information:**

The online version contains supplementary material available at 10.1007/s00441-025-04013-w.

## Introduction

Autophagy is a major intracellular degradation system in which portions of the cytoplasm, including organelles, are sequestered by autophagic isolation membranes, termed phagophores, to form autophagosomes. The outer membranes of autophagosomes subsequently fuse with lysosomes to form autolysosomes in which the internal contents are degraded. This process plays a crucial role in maintaining cellular homeostasis, particularly under conditions of stress or damage (see review by Mizushima and Komatsu, 2011; Yamamoto et al., 2023). Autophagy can be classified into several selective forms based on the target substrate.

There is a growing body of evidence regarding the origin and formation of phagophores. In vitro studies reported that autophagosomes often originate from the endoplasmic reticulum (ER), particularly at ER-mitochondria contact sites in mammalian cells (Hamasaki et al., 2013), although some studies suggested that other cellular compartments, including the Golgi apparatus, the plasma membrane, and mitochondria, make minor contributions to autophagosome formation (Lamb et al., 2013). Expansion of phagophores is associated with ER subdomains such as the omegasome and tubular structures that connect the ER to the edges of phagophores (Uemura et al., 2014). Furthermore, morphological studies using cell culture systems have shown physical continuity between the ER membrane and phagophores (Biazik et al., 2015; Bieber et al., 2022; Hayashi-Nishino et al., 2010; Ylä-Anttila et al., 2009). However, the structural relationship between the ER membrane and phagophores in vivo has not been reported.

The ER is an essential organelle responsible for the synthesis, folding, assembly, and transport of proteins (see review by Kaufman, 1999). Properly folded proteins are either packaged into secretory granules or transported to cell membranes via the Golgi apparatus (Braakman and Bulleid, 2011). Only proteins destined for export from the cell are packaged in this way. In contrast, misfolded or unfolded proteins accumulate in the ER, leading to ER stress (Wang and Kaufman, 2012). To alleviate ER stress, cells activate the unfolded protein response (Ron and Walter, 2007). ER-phagy, one form of selective autophagy, is induced as a compensatory and protective mechanism under ER stress conditions to remove misfolded/unfolded proteins and damaged ER structures (see review by Chino and Mizushima, 2023; Mochida and Nakatogawa, 2022).

Arginine vasopressin (AVP), an antidiuretic hormone, is synthesized in magnocellular neurons of the supraoptic nuclei (SON) and paraventricular nuclei (PVN) of the hypothalamus (Bisset and Chowdrey, 1988). The *AVP* gene encodes a signal peptide, AVP, the AVP carrier protein neurophysin II (NPII), and a glycoprotein known as copeptin (Land et al., 1982). Following cleavage of the signal peptide, prepro-AVP is processed into pro-AVP, which folds into its native conformation in the ER before being packaged into secretory granules. During transport to the posterior pituitary, pro-AVP is further processed, and AVP is ultimately released into the bloodstream in response to changes in plasma osmolality and blood pressure (Brownstein et al., 1980; Burbach et al., 2001).

Familial neurohypophysial diabetes insipidus (FNDI) is an autosomal dominant disorder caused by mutations in the *AVP* gene, particularly within the NPII coding region (see review by Arima et al., 2016; Christensen and Rittig, 2006). We previously generated a knock-in mouse model of FNDI by introducing a disease-causing NPII mutation into the *AVP* gene locus. These heterozygous mice exhibit phenotypes that closely resemble those of human FNDI patients, including polyuria, polydipsia, and decreased urine osmolality (Hayashi et al., 2009). Electron microscopy of AVP neurons from these mice showed that mutant aggregates accumulate in a specific rough ER subdomain known as the ER-associated compartment (ERAC) (Hagiwara et al., 2014; Miyata et al., 2020). Notably, as these mice aged, or became dehydrated following water deprivation, these aggregates spread throughout the dilated ER lumen, triggering ER-phagy in response to elevated ER stress (Hagiwara et al., 2014; Hagiwara et al., 2019).

In the present study, we used serial block-face scanning electron microscopy (SBF-SEM) to investigate the structural relationships between phagophores, ER membranes, and mutant protein aggregates within the dilated ER of AVP neurons from FNDI mice.

## Materials and methods

### Animals

FNDI mice heterozygous for the mutant *Avp* gene (Cys98stop) were generated previously (Hayashi et al., 2009). All FNDI mice in the present study were backcrossed over 15 generations onto the C57BL/6J background. Mice were maintained under controlled conditions (23.0 ± 0.5 °C, Lights on 09:00 to 21:00), and male mice were used in the experiments. All procedures were approved by the Animal Experimentation Committee of the Nagoya University Graduate School of Medicine (registration number M250067-003) and performed in accordance with institutional guidelines for animal care and use.

### Water deprivation

Two-month-old male FNDI mice were subjected to intermittent water deprivation for 4 weeks that consisted of repeated cycles of continuous water deprivation for 48 h followed by a recovery period of 5 days with free access to water, as reported previously (Kurimoto et al., 2021). Mice were decapitated immediately after the last 48 h of water deprivation.

### SBF-SEM

SBF-SEM analyses were performed as described previously with slight modifications (Matsumoto et al., 2019). Briefly, FNDI mice subjected to intermittent water deprivation for 4 weeks were deeply anesthetized and transcardially perfused with 4% paraformaldehyde and 2.5% glutaraldehyde in 0.1 M phosphate buffer (pH 7.4). After fixation, the brains were immediately removed and immersed overnight at 4 °C in the same fixative. Brains were cut into 100 µm sections on a Vibratome (VT1200 S; Leica Biosystems, Wetzlar, Germany). Tissues were treated with 2% OsO_4_ in 1.5% K_4_[Fe(CN)_6_] for 1 h at 4 °C, and subsequently with 1% thiocarbohydrazide for 20 min, and 2% OsO_4_ for 30 min at room temperature. Thereafter, the tissues were treated with 1% uranyl acetate at 4 °C overnight and then with lead aspartate solution, produced by adding 20 mM lead nitrate (NisshinEM) to 30 mM L-aspartic acid (Nacalai Tesque) in distilled water prior to adjusting the pH to 5.5, for 2 h at 50 °C. The tissues were dehydrated in a graded series of ethanol solutions (60%, 80%, 90%, 95%) and then with pure acetone before embedding in Durcupan resin containing Ketjen black powder (5%) for 48 h at 60 °C to ensure polymerization. SBF-SEM observation of the SON was performed using a SigmaVP scanning electron microscope (Carl Zeiss) equipped with a 3View in-chamber ultramicrotome system (Gatan). Serial image sequences were acquired at 50 nm steps at a resolution of 5.0 nm per pixel. The serial images were processed using TrakEM2 (Cardona et al., 2012) in ImageJ with Fiji plugins (http://fiji.sc/) for binning, contrast adjustment, and alignment. Manual segmentation and three-dimensional reconstruction were performed using Microscopy Image Browser (version 2.70, http://mib.helsinki.fi) (Belevich et al., 2016), VAST Lite (version 1.4.0) (Berger et al., 2018), and Amira software (version 2020.1, FEI Visualization Science Group, Hillsboro, OR, USA). The segmentation was supported by UNI-EM, a unified environment for CNN-based automated segmentation of EM images (version 0.92) (Urakubo et al., 2019), for generation of prediction maps of phagophores and protein aggregates. The training data production and subsequent manual proofreading were performed using Microscopy Image Browser, VAST Lite, and Amira.

## Results

**Fig. 1:**
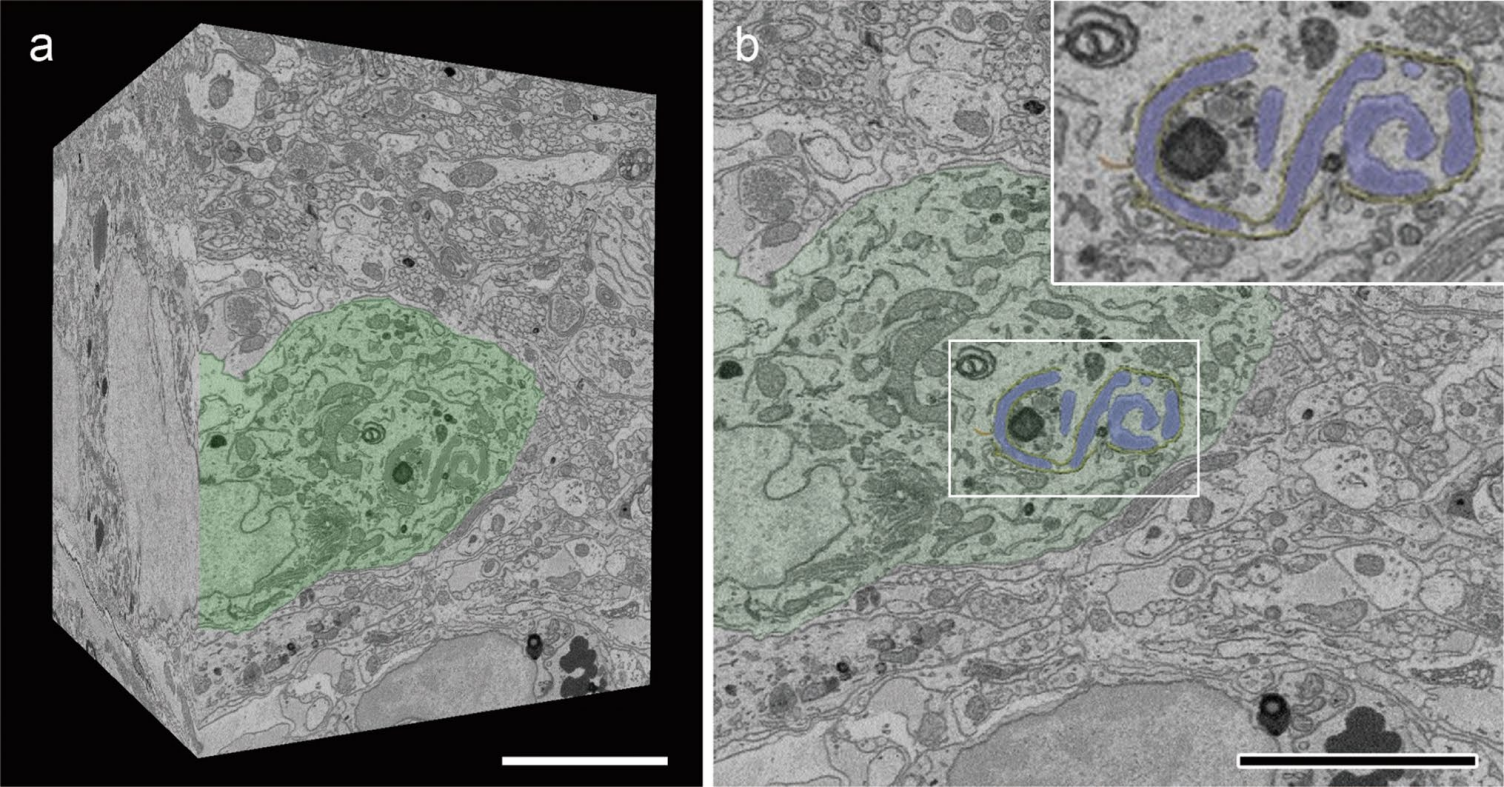
SBF-SEM analysis of the SON in FNDI mice. **a** Three-dimensional reconstruction of a brain volume from serial electron microscopic images of the SON in FNDI mice. Part of an AVP neuron (green) is shown on the surface of the imaged volume. Scale bar: 5 µm. **b** The AVP neuron (green) contains mutant protein aggregates within the dilated ER (blue), surrounding phagophores (yellow) and intact rough ER (red). The boxed region is shown at higher magnification. Scale bar: 5 µm

**Fig. 2:**
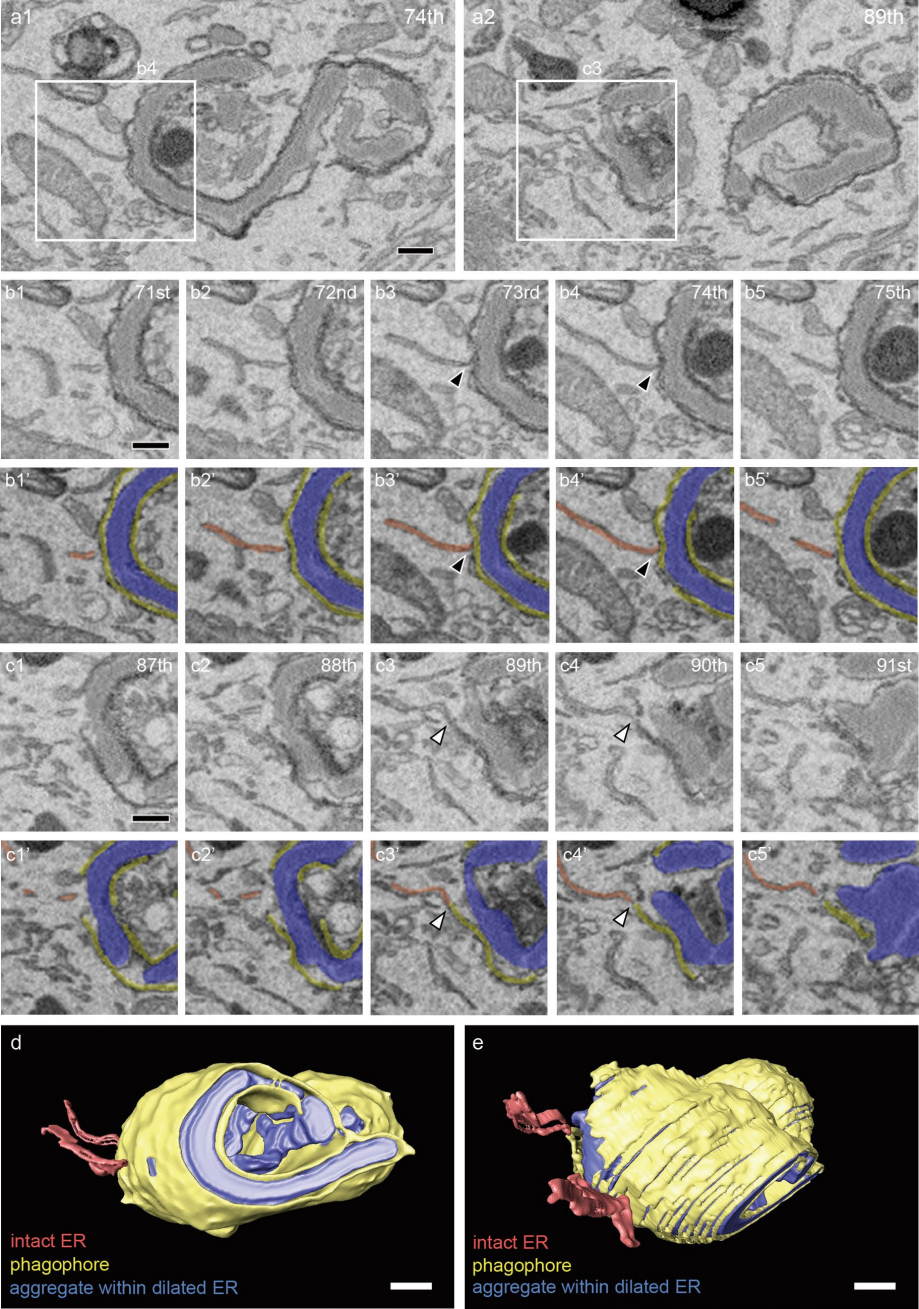
Connection between phagophores and the intact ER membrane in the AVP neuron is shown in Fig. 1. **a**–**c** Serial images showing mutant protein aggregates within the dilated ER. The boxed areas in a1 and a2 are enlarged in b4 and c3, respectively. Magnified serial images (b1-5 and c1-5) reveal the physical continuity between phagophores and the intact ER membrane, indicated by black and white arrowheads. Corresponding colorized images (b1’−5’ and c1’−5’) highlight aggregates within the dilated ER in blue, phagophores in yellow and the intact ER in red. **d** and **e** Three-dimensional reconstructions of aggregates within the dilated ER (blue), phagophores (yellow), and the intact ER (red). Numbers in the upper right corners indicate the slice number in the electron microscopic image stack. Scale bars: 500 nm. See also Videos S1 and S2

In three-dimensional electron microscopic analyses using SBF-SEM, aggregates of mutant NPII were identified by the presence of dilated continuous cisternae that appeared as electron-dense areas in the lumen of AVP neurons in the SON of FNDI mice that were subjected to 4 weeks of intermittent water deprivation (Fig. 1). The ERAC structure was disrupted and had a different appearance from the large spherical structure observed in AVP neurons from FNDI mice that were not subjected to water deprivation (Miyata et al., 2020), and mutant NPII aggregates were dispersed throughout the dilated ER lumen (Fig. 1b). Phagophores, characterized by spreading cisternae with narrow lumens and electron-dense membranes (Arai and Waguri, 2019), were observed emerging and partially surrounding regions of the dilated ER (Figs. 1b and 2a). Careful tracing of serial images revealed that phagophores were physically continuous with the intact ER membrane, which was characterized by tubular or flattened cisternae lacking luminal NPII aggregates, and extended throughout the cytoplasm (Fig. 2b–e, and Supplementary Videos S1 and S2).

## Discussion

Animal models in which abnormal proteins accumulate in the ER and trigger autophagy are limited. In the present study, we used FNDI mice as an in vivo model in which ER-phagy was induced by ER stress (Hagiwara et al., 2019; Miyata et al., 2020). Using this model, we applied SBF-SEM to demonstrate that phagophores are physically connected to the intact ER membrane in AVP neurons from FNDI mice. While previous in vitro studies have suggested that phagophores may originate from specific domains of the ER (Hayashi-Nishino et al., 2009; Nakatogawa, 2020; Nishimura et al., 2017), to our knowledge, this is the first in vivo demonstration of the structural continuity between phagophores and the ER membrane. Future studies will be required to clarify whether the ER also serves as the membrane source for phagophore formation for forms of selective autophagy other than ER-phagy or non-selective bulk autophagy pathways.

Previous morphological studies reported that phagophore membranes display high electron density when visualized in samples prepared using reduced osmium, although the exact molecular composition of these dense membranes remains unknown (Arai and Waguri, 2019; Biazik et al., 2015). Consistently, in our previous study (Miyata et al., 2020), we observed electron-dense membranous structures surrounding large ERACs filled with mutant NPII aggregates in AVP neurons from FNDI mice. The distribution of these electron-dense structures was consistent with microtubule-associated protein 1 light chain 3 (LC3)-positive phagophores (Miyata et al., 2020). The SBF-SEM imaging method with reduced osmium used in the present study enhances contrast, particularly in membranes such as those in phagophores and autophagosomes, and reduces the distance between the double-membrane structures. SBF-SEM imaging is also advantageous for identifying multiple contact sites between the phagophore and surrounding organelles, such as the ER, and proved especially useful for observing the continuity between phagophores and ER membranes in segmentation and three-dimensional reconstruction in our analysis.

In conclusion, our findings provide in vivo ultrastructural evidence that the ER membrane serves as the origin of phagophore formation during ER-phagy.

## Supplementary Information

Three-dimensional reconstructions showing the connection between phagophores and the intact ER membrane. The intact ER, phagophores, and protein aggregates within the dilated ER are shown in orange, yellow, and blue, respectively.

ESM 1 (13. 5 MB MPEG)

ESM 2 (27.4 MB MPEG)

## Data Availability

All data generated in this study will be available from the corresponding author upon request.

## References

[CR1] Arai R, Waguri S (2019) Improved electron microscopy fixation methods for tracking autophagy-associated membranes in cultured mammalian cells. Methods Mol Biol 1880:211–221 30610699 10.1007/978-1-4939-8873-0_13

[CR2] Arima H, Azuma Y, Morishita Y, Hagiwara D (2016) Central diabetes insipidus. Nagoya J Med Sci 78:349–358 28008190 10.18999/nagjms.78.4.349 PMC5159460

[CR3] Belevich I, Joensuu M, Kumar D, Vihinen H, Jokitalo E (2016) Microscopy image browser: a platform for segmentation and analysis of multidimensional datasets. PLoS Biol 14:e1002340 26727152 10.1371/journal.pbio.1002340 PMC4699692

[CR4] Berger DR, Seung HS, Lichtman JW (2018) Vast (volume annotation and segmentation tool): efficient manual and semi-automatic labeling of large 3d image stacks. Front Neural Circuits 12:88 30386216 10.3389/fncir.2018.00088 PMC6198149

[CR5] Biazik J, Ylä-Anttila P, Vihinen H, Jokitalo E, Eskelinen EL (2015) Ultrastructural relationship of the phagophore with surrounding organelles. Autophagy 11:439–451 25714487 10.1080/15548627.2015.1017178 PMC4502653

[CR6] Bieber A, Capitanio C, Erdmann PS, Fiedler F, Beck F, Lee CW, Li D, Hummer G et al (2022) In situ structural analysis reveals membrane shape transitions during autophagosome formation. Proc Natl Acad Sci U S A 119:e2209823119 36122245 10.1073/pnas.2209823119 PMC9522377

[CR7] Bisset GW, Chowdrey HS (1988) Control of release of vasopressin by neuroendocrine reflexes. Q J Exp Physiol 73:811–872 2907166 10.1113/expphysiol.1988.sp003223

[CR8] Braakman I, Bulleid NJ (2011) Protein folding and modification in the mammalian endoplasmic reticulum. Annu Rev Biochem 80:71–99 21495850 10.1146/annurev-biochem-062209-093836

[CR9] Brownstein MJ, Russell JT, Gainer H (1980) Synthesis, transport, and release of posterior pituitary hormones. Science 207:373–378 6153132 10.1126/science.6153132

[CR10] Burbach JP, Luckman SM, Murphy D, Gainer H (2001) Gene regulation in the magnocellular hypothalamo-neurohypophysial system. Physiol Rev 81:1197–1267 11427695 10.1152/physrev.2001.81.3.1197

[CR11] Cardona A, Saalfeld S, Schindelin J, Arganda-Carreras I, Preibisch S, Longair M, Tomancak P, Hartenstein V et al (2012) TrakEM2 software for neural circuit reconstruction. PLoS ONE 7:e38011 22723842 10.1371/journal.pone.0038011 PMC3378562

[CR12] Chino H, Mizushima N (2023) ER-phagy: quality and quantity control of the endoplasmic reticulum by autophagy. Cold Spring Harb Perspect Biol. 10.1101/cshperspect.a041256 35940904 10.1101/cshperspect.a041256 PMC9808580

[CR13] Christensen JH, Rittig S (2006) Familial neurohypophyseal diabetes insipidus–an update. Semin Nephrol 26:209–223 16713494 10.1016/j.semnephrol.2006.03.003

[CR14] Hagiwara D, Arima H, Morishita Y, Wenjun L, Azuma Y, Ito Y, Suga H, Goto M et al (2014) Arginine vasopressin neuronal loss results from autophagy-associated cell death in a mouse model for familial neurohypophysial diabetes insipidus. Cell Death Dis 5:e1148 24675466 10.1038/cddis.2014.124 PMC3973212

[CR15] Hagiwara D, Grinevich V, Arima H (2019) A novel mechanism of autophagy-associated cell death of vasopressin neurons in familial neurohypophysial diabetes insipidus. Cell Tissue Res 375:259–266 29961215 10.1007/s00441-018-2872-4

[CR16] Hamasaki M, Furuta N, Matsuda A, Nezu A, Yamamoto A, Fujita N, Oomori H, Noda T et al (2013) Autophagosomes form at ER-mitochondria contact sites. Nature 495:389–393 23455425 10.1038/nature11910

[CR17] Hayashi M, Arima H, Ozaki N, Morishita Y, Hiroi M, Ozaki N, Nagasaki H, Kinoshita N et al (2009) Progressive polyuria without vasopressin neuron loss in a mouse model for familial neurohypophysial diabetes insipidus. Am J Physiol Regul Integr Comp Physiol 296:R1641-1649 19297548 10.1152/ajpregu.00034.2009

[CR18] Hayashi-Nishino M, Fujita N, Noda T, Yamaguchi A, Yoshimori T, Yamamoto A (2009) A subdomain of the endoplasmic reticulum forms a cradle for autophagosome formation. Nat Cell Biol 11:1433–1437 19898463 10.1038/ncb1991

[CR19] Hayashi-Nishino M, Fujita N, Noda T, Yamaguchi A, Yoshimori T, Yamamoto A (2010) Electron tomography reveals the endoplasmic reticulum as a membrane source for autophagosome formation. Autophagy 6:301–303 20104025 10.4161/auto.6.2.11134

[CR20] Kaufman RJ (1999) Stress signaling from the lumen of the endoplasmic reticulum: coordination of gene transcriptional and translational controls. Genes Dev 13:1211–1233 10346810 10.1101/gad.13.10.1211

[CR21] Kurimoto J, Takagi H, Miyata T, Hodai Y, Kawaguchi Y, Hagiwara D, Suga H, Kobayashi T et al (2021) Deficiency of WFS1 leads to the impairment of AVP secretion under dehydration in male mice. Pituitary 24:582–588 33666833 10.1007/s11102-021-01135-6

[CR22] Lamb CA, Yoshimori T, Tooze SA (2013) The autophagosome: origins unknown, biogenesis complex. Nat Rev Mol Cell Biol 14:759–774 24201109 10.1038/nrm3696

[CR23] Land H, Schütz G, Schmale H, Richter D (1982) Nucleotide sequence of cloned cDNA encoding bovine arginine vasopressin-neurophysin II precursor. Nature 295:299–303 6276766 10.1038/295299a0

[CR24] Matsumoto M, Sawada M, García-González D, Herranz-Pérez V, Ogino T, Bang Nguyen H, Quynh Thai T, Narita K et al (2019) Dynamic changes in ultrastructure of the primary cilium in migrating neuroblasts in the postnatal brain. J Neurosci 39:9967–9988 31685650 10.1523/JNEUROSCI.1503-19.2019 PMC6978947

[CR25] Miyata T, Hagiwara D, Hodai Y, Miwata T, Kawaguchi Y, Kurimoto J, Ozaki H, Mitsumoto K et al (2020) Degradation of mutant protein aggregates within the endoplasmic reticulum of vasopressin neurons. iScience 23:101648 33103081 10.1016/j.isci.2020.101648 PMC7578753

[CR26] Mizushima N, Komatsu M (2011) Autophagy: renovation of cells and tissues. Cell 147:728–741 22078875 10.1016/j.cell.2011.10.026

[CR27] Mochida K, Nakatogawa H (2022) ER-phagy: selective autophagy of the endoplasmic reticulum. EMBO Rep 23:e55192 35758175 10.15252/embr.202255192 PMC9346472

[CR28] Nakatogawa H (2020) Mechanisms governing autophagosome biogenesis. Nat Rev Mol Cell Biol 21:439–458 32372019 10.1038/s41580-020-0241-0

[CR29] Nishimura T, Tamura N, Kono N, Shimanaka Y, Arai H, Yamamoto H, Mizushima N (2017) Autophagosome formation is initiated at phosphatidylinositol synthase-enriched ER subdomains. EMBO J 36:1719–1735 28495679 10.15252/embj.201695189 PMC5470044

[CR30] Ron D, Walter P (2007) Signal integration in the endoplasmic reticulum unfolded protein response. Nat Rev Mol Cell Biol 8:519–529 17565364 10.1038/nrm2199

[CR31] Uemura T, Yamamoto M, Kametaka A, Sou YS, Yabashi A, Yamada A, Annoh H, Kametaka S et al (2014) A cluster of thin tubular structures mediates transformation of the endoplasmic reticulum to autophagic isolation membrane. Mol Cell Biol 34:1695–1706 24591649 10.1128/MCB.01327-13 PMC3993601

[CR32] Urakubo H, Bullmann T, Kubota Y, Oba S, Ishii S (2019) UNI-EM: an environment for deep neural network-based automated segmentation of neuronal electron microscopic images. Sci Rep 9:19413 31857624 10.1038/s41598-019-55431-0 PMC6923391

[CR33] Wang S, Kaufman RJ (2012) The impact of the unfolded protein response on human disease. J Cell Biol 197:857–867 22733998 10.1083/jcb.201110131 PMC3384412

[CR34] Yamamoto H, Zhang S, Mizushima N (2023) Autophagy genes in biology and disease. Nat Rev Genet 24:382–400 36635405 10.1038/s41576-022-00562-w PMC9838376

[CR35] Ylä-Anttila P, Vihinen H, Jokitalo E, Eskelinen EL (2009) 3D tomography reveals connections between the phagophore and endoplasmic reticulum. Autophagy 5:1180-1185 10.4161/auto.5.8.10274 19855179

